# Impact of methodological choices in comparative effectiveness studies: application in natalizumab versus fingolimod comparison among patients with multiple sclerosis

**DOI:** 10.1186/s12874-022-01623-8

**Published:** 2022-05-30

**Authors:** M. Lefort, S. Sharmin, J. B. Andersen, S. Vukusic, R. Casey, M. Debouverie, G. Edan, J. Ciron, A. Ruet, J. De Sèze, E. Maillart, H. Zephir, P. Labauge, G. Defer, C. Lebrun-Frenay, T. Moreau, E. Berger, P. Clavelou, J. Pelletier, B. Stankoff, O. Gout, E. Thouvenot, O. Heinzlef, A. Al-Khedr, B. Bourre, O. Casez, P. Cabre, A. Montcuquet, A. Wahab, J. P. Camdessanché, A. Maurousset, H. Ben Nasr, K. Hankiewicz, C. Pottier, N. Maubeuge, D. Dimitri-Boulos, C. Nifle, D. A. Laplaud, D. Horakova, E. K. Havrdova, R. Alroughani, G. Izquierdo, S. Eichau, S. Ozakbas, F. Patti, M. Onofrj, A. Lugaresi, M. Terzi, P. Grammond, F. Grand’Maison, B. Yamout, A. Prat, M. Girard, P. Duquette, C. Boz, M. Trojano, P. McCombe, M. Slee, J. Lechner-Scott, R. Turkoglu, P. Sola, D. Ferraro, F. Granella, V. Shaygannejad, J. Prevost, D. Maimone, O. Skibina, K. Buzzard, A. Van der Walt, R. Karabudak, B. Van Wijmeersch, T. Csepany, D. Spitaleri, S. Vucic, N. Koch-Henriksen, F. Sellebjerg, P. S. Soerensen, C. C. Hilt Christensen, P. V. Rasmussen, M. B. Jensen, J. L. Frederiksen, S. Bramow, H. K. Mathiesen, K. I. Schreiber, H. Butzkueven, M. Magyari, T. Kalincik, E. Leray

**Affiliations:** 1 Arènes - UMR 6051, RSMS (Recherche sur les Services et Management en Santé) - U 1309, Univ Rennes, EHESP, CNRS, Inserm, Rennes, France; 2 Univ Rennes, CHU Rennes, Investigation Clinique de Rennes)], CIC 1414 [(Centre d, 35000 InsermRennes, France; 3 Department of Medicine, University of Melbourne, Melbourne, Australia; 4 Melbourne MS Centre, Department of Neurology, Royal Melbourne Hospital, Melbourne, Australia; 5 Department of Neurology, The Danish Multiple Sclerosis Registry, Copenhagen University Hospital, Rigshospitalet Glostrup, Denmark; 6 Service de Neurologie, Sclérose en Plaques, Pathologies de La Myéline Et Neuro-Inflammation, Hôpital Neurologique Pierre Wertheimer, Hospices Civils de Lyon, 69677 Lyon/Bron, France; 7 Centre Des Neurosciences de Lyon, UMR5292, Observatoire Français de La Sclérose en Plaques, INSERM, 1028 et CNRS, 69003 Lyon, France; 8 Université, Claude Bernard Lyon 1, Faculté de médecine Lyon Est, 69000 Lyon, France; 9 Eugene Devic EDMUS Foundation, 69677 Lyon/Bron, France; 10 Centre Hospitalier Régional Universitaire de Nancy, Hôpital Central, Service de neurologie, Nancy, France; 11 Centre Hospitalier Universitaire de Rennes, Hôpital Pontchaillou, Service de neurologie, Rennes, France; 12 Centre Hospitalier Universitaire de Toulouse, Hôpital Purpan, CRC-SEP, Département de neurologie, Toulouse, France; 13 Centre Hospitalier Universitaire de Bordeaux, Hôpital Pellegrin, Service de neurologie, Bordeaux, France; 14 Service des maladies inflammatoires du système nerveux – neurologie, centre d’investigation clinique de Strasbourg, Hôpitaux Universitaire de Strasbourg, Hôpital de Hautepierre, INSERM 1434, Strasbourg, France; 15 Assistance Publique Des Hôpitaux de Paris, Hôpital de La Pitié-Salpêtrière, Service de neurologie, Paris, France; 16 Centre Hospitalier Universitaire de Lille, Hôpital Salengro, Service de neurologie D, Lille, France; 17 Centre Hospitalier Universitaire de Montpellier, Hôpital Gui de Chauliac, Service de neurologie, Montpellier, France; 18 Centre Hospitalier Universitaire de Caen Normandie, Hôpital Côte de Nacre, Service de neurologie, Caen, France; 19 Centre Hospitalier Universitaire de Nice, UR2CA-URRIS,, Université Nice Côte d’Azur, Hôpital, Pasteur 2, Service de neurologie, Nice, France; 20 Centre Hospitalier Universitaire Dijon Bourgogne, Hôpital François Mitterrand, Maladies Inflammatoires du Système Nerveux Et Neurologie Générale, Service de neurologie, Dijon, France; 21 Centre Hospitalier Régional Universitaire de Besançon, Hôpital Jean Minjoz, Service de neurologie, Besançon, France; 22 Centre Hospitalier Universitaire de Clermont-Ferrand, Hôpital Gabriel-Montpied, Service de neurologie, Clermont-Ferrand, France; 23 Service de Neurologie, Aix Marseille Univ, APHM, Hôpital de La Timone, Pôle de Neurosciences Cliniques, 13005 Marseille, France; 24 Assistance Publique Des Hôpitaux de Paris, Hôpital Saint-Antoine, Service de neurologie, Paris, France; 25 Fondation Adolphe de Rothschild de L’œil Et du Cerveau, Service de neurologie, Paris, France; 26 Centre Hospitalier Universitaire de Nîmes, Hôpital Carémeau, Service de neurologie, Nîmes, France; 27 Centre Hospitalier Intercommunal de Poissy Saint-Germain-en-Laye, Service de neurologie, Poissy, France; 28 Centre Hospitalier Universitaire d’Amiens Picardie, Site sud, Service de neurologie, Amiens, France; 29 Rouen University Hospital, 76000 Rouen, France; 30 Centre Hospitalier Universitaire Grenoble-Alpes, Site nord, Service de neurologie, Grenoble/La Tronche, France; 31 Centre Hospitalier Universitaire de Martinique, Hôpital Pierre Zobda-Quitman, Service de neurologie, Fort-de-France, France; 32 Centre Hospitalier Universitaire Limoges, Hôpital Dupuytren, Service de neurologie, Limoges, France; 33 Assistance Publique Des Hôpitaux de Paris, Hôpital Henri Mondor, Service de neurologie, Créteil, France; 34 Centre Hospitalier Universitaire de Saint-Étienne, Hôpital Nord, Service de neurologie, Saint-Étienne, France; 35 Centre Hospitalier Régional Universitaire de Tours, Hôpital Bretonneau, Service de neurologie, Tours, France; 36 Centre Hospitalier Sud Francilien, Service de neurologie, Corbeil-Essonnes, France; 37 Centre Hospitalier de Saint-Denis, Hôpital Casanova, Service de neurologie, Saint-Denis, France; 38 Centre Hospitalier de Pontoise, Service de neurologie, Pontoise, France; 39 Centre Hospitalier Universitaire de Poitiers, Site de La Milétrie, Service de neurologie, Poitiers, France; 40 Assistance Publique Des Hôpitaux de Paris, Hôpital Bicêtre, Service de neurologie, Le Kremlin-Bicêtre, France; 41 Centre Hospitalier de Versailles, Hôpital André-Mignot, Service de neurologie, Le Chesnay, France; 42 CHU de Nantes, Service de Neurologie & CIC015 INSERM, 44093 Nantes, France; 43 INSERM CR1064, 44000 Nantes, France; 44 Department of Neurology and Center of Clinical Neuroscience, First Faculty of Medicine, Charles University in Prague and General University Hospital, Prague, Czech Republic; 45 Division of Neurology, Department of Medicine, Amiri Hospital, Sharq, Kuwait; 46 Hospital Universitario Virgen Macarena, Seville, Spain; 47 Dokuz Eylul University, Konak/Izmir, Turkey; 48 GF Ingrassia Department, University of Catania, Catania, Italy; 49 Policlinico G Rodolico, Catania, Italy; 50 Department of Neuroscience, Imaging, and Clinical Sciences, University G. d’Annunzio, Chieti, Italy; 51 Dipartimento Di Scienze Biomediche E Neuromotorie, Università Di Bologna, Bologna, Italy; 52 IRCCS Istituto Delle Scienze Neurologiche Di Bologna, Bologna, Italy; 53 Medical Faculty, 19 Mayis University, Samsun, Turkey; 54 CISSS Chaudiere-Appalache, Levis, Canada; 55 Neuro Rive-Sud, Longueuil, QC Canada; 56 Nehme and Therese Tohme Multiple Sclerosis Center, American University of Beirut Medical Center, Beirut, Lebanon; 57 Hopital Notre Dame, Montreal, Canada; 58 CHUM and Universite de Montreal, Montreal, Canada; 59 KTU Medical Faculty Farabi Hospital, Trabzon, Turkey; 60 Department of Basic Medical Sciences, Neuroscience and Sense Organs, University of Bari, Bari, Italy; 61 University of Queensland, Brisbane, Australia; 62 Royal Brisbane and Women’s Hospital, Herston, Australia; 63 Flinders University, Adelaide, Australia; 64 School of Medicine and Public Health, University Newcastle, Newcastle, Australia; 65 Department of Neurology, John Hunter Hospital, Hunter New England Health, Newcastle, Australia; 66 Haydarpasa Numune Training and Research Hospital, Istanbul, Turkey; 67 Department of Neuroscience, Azienda Ospedaliera Universitaria, Modena, Italy; 68 Department of Medicine and Surgery, University of Parma, Parma, Italy; 69 Department of Emergency and General Medicine, Parma University Hospital, Parma, Italy; 70 Isfahan University of Medical Sciences, Isfahan, Iran; 71 CSSS Saint-Jérôme, Saint-Jerome, Canada; 72 Garibaldi Hospital, Catania, Italy; 73 Monash University, Melbourne, Australia; 74 Hacettepe University, Ankara, Turkey; 75 Rehabilitation and MS-Centre Overpelt and Hasselt University, Hasselt, Belgium; 76 Department of Neurology, Faculty of Medicine, University of Debrecen, Debrecen, Hungary; 77 Azienda Ospedaliera Di Rilievo Nazionale San Giuseppe Moscati Avellino, Avellino, Italy; 78 Westmead Hospital, Sydney, Australia; 79 Department of Clinical Epidemiology, Aarhus University Hospital Aarhus, Aarhus, Denmark; 80 Danish Multiple Sclerosis Centre, Department of Neurology, Copenhagen University Hospital, Rigshospitalet Glostrup, 2600 Glostrup, Denmark; 81 Department of Neurology, Aalborg University Hospital, Multiple Sclerosis Unit, Aalborg, Denmark; 82 Aarhus University Hospital, Neurology, PJJ Boulevard, DK-8200 Aarhus N, Denmark; 83 Department of Neurology, University Hospital of Northern Sealand, Copenhagen, Denmark; 84 Department of Clinical Medicine, University of Copenhagen, Copenhagen, Denmark; 85 Department of Neurology, Copenhagen University Hospital Herlev, Copenhagen, Denmark; 86 Central Clinical School, Monash University, Melbourne, Australia; 87 Department of Neurology, The Alfred Hospital, Melbourne, Australia; 88 Department of Neurology, Box Hill Hospital, Monash University, Melbourne, Australia

**Keywords:** Effectiveness, Multiple sclerosis, Propensity score, Indication bias, Causal contrasts, Censoring, Positivity assumption

## Abstract

**Background:**

Natalizumab and fingolimod are used as high-efficacy treatments in relapsing–remitting multiple sclerosis. Several observational studies comparing these two drugs have shown variable results, using different methods to control treatment indication bias and manage censoring. The objective of this empirical study was to elucidate the impact of methods of causal inference on the results of comparative effectiveness studies.

**Methods:**

Data from three observational multiple sclerosis registries (MSBase, the Danish MS Registry and French OFSEP registry) were combined. Four clinical outcomes were studied. Propensity scores were used to match or weigh the compared groups, allowing for estimating average treatment effect for treated or average treatment effect for the entire population. Analyses were conducted both in intention-to-treat and per-protocol frameworks. The impact of the positivity assumption was also assessed.

**Results:**

Overall, 5,148 relapsing–remitting multiple sclerosis patients were included. In this well-powered sample, the 95% confidence intervals of the estimates overlapped widely. Propensity scores weighting and propensity scores matching procedures led to consistent results. Some differences were observed between average treatment effect for the entire population and average treatment effect for treated estimates. Intention-to-treat analyses were more conservative than per-protocol analyses. The most pronounced irregularities in outcomes and propensity scores were introduced by violation of the positivity assumption.

**Conclusions:**

This applied study elucidates the influence of methodological decisions on the results of comparative effectiveness studies of treatments for multiple sclerosis. According to our results, there are no material differences between conclusions obtained with propensity scores matching or propensity scores weighting given that a study is sufficiently powered, models are correctly specified and positivity assumption is fulfilled.

**Supplementary Information:**

The online version contains supplementary material available at 10.1186/s12874-022-01623-8.

## Background

Natalizumab [1, 2] and fingolimod [3, 4] are two high-efficacy treatments used in Relapsing Remitting Multiple Sclerosis (RRMS) patients. Interestingly, the comparative effectiveness studies comparing these therapies showed results that were somewhat inconsistent [5–9]. In particular, we focus on three studies which used data from three multiple sclerosis (MS) registries, with differences in methods and conclusions [5–7]. We have already shown that some of this variability can be attributed to differences between the study populations [10, 11] ﻿. In the present work, we focus on the impact of methodological choices on the results—in particular, the methods used to control treatment indication bias and to manage censoring in time-to-event analysis.

In the absence of randomized clinical trials, many decisions need to be made to conduct observational studies. In the framework of “target trial”, developed by Hernan and Robins, we will focus on two protocol components, first, the assignment procedure and, second, the causal contrast [12]. First, to emulate the random assignment, we need to adjust for all known confounders [12]. Propensity score (PS), utilized in several ways, is a popular instrument used to control indication bias effect on the results of comparisons of intervention [13, 14]. The studies in the Danish MS Registry and MSBase used PS matching [6, 7] while the study in OFSEP used PS weighting [5]. Second, attrition bias and informative censoring result from systematic differences in the follow-up duration between cohorts. Two causal contrasts, per-protocol and intention-to-treat, were considered to evaluate follow-up information. While the per-protocol framework includes only outcomes that were recorded while patients were exposed to the relevant intervention, intention-to-treat framework mitigates the risk of informed censoring, which is of particular importance where clinical outcomes between interventions are delayed [12, 15]. The per-protocol framework was originally used in the studies in the Danish MS Registry and MSBase [6, 7] while the intention-to-treat framework was used in the OFSEP study [5]. Moreover, the study in MSBase used pairwise censoring that consists of censoring data within each PS matched pair to the shorter of the recorded follow-up times within the pair, in order to balance the analysed follow-up time between the groups [16].

The objective of this empirical study is to elucidate the influence of methodological decisions on the results of a comparison of two potent interventions, using the example of natalizumab and fingolimod among patients with MS and combined data from three large clinical registries [5–7].

## Methods

### Data source

This study is a result of a collaborative project [11, 17]. Longitudinal demographic and clinical data were extracted from MSBase on 15^th^ of May 2018 [18, 19]. The Danish MS Registry cohort included all patients treated with natalizumab or fingolimod from 1^st^ of July, 2011 when fingolimod became available in Denmark, until 1^st^ of March, 2018 [20, 21]. The OFSEP cohort included data from 27 French university hospitals extracted from the European Database for Multiple Sclerosis (EDMUS) software in July 2014 [22]. No patient from OFSEP was recorded in MSBase. Some Danish patients who were recorded both in MSBase and Danish MS Registry (2% of Danish MS Registry) have been excluded from MSBase and only considered in the Danish MS Registry.

### Eligibility criteria

All patients were diagnosed with RRMS. The required disability follow-up consisted of: a recorded visit with Expanded Disability Status Scale (EDSS)[23] score assessment within six months before treatment initiation (the baseline visit), two post-baseline visits with EDSS at least six months apart, and at least one on-treatment visit.

### Interventions

Treatments of interest were the first exposure to natalizumab or fingolimod on or after 1^st^ January 2011 and continued for a minimum of three months. Patients who participated in randomized trials or patients treated with off-label treatment (cyclophosphamide), or with therapies known to have extended duration of effect [24–26] (mitoxantrone, alemtuzumab, cladribine, daclizumab, rituximab, ocrelizumab) before the study therapy were excluded. Each patient could contribute only once to the follow-up analysis. When multiple eligible treatment starts were recorded, the earliest treatment was considered.

### Outcomes

Four outcomes were evaluated to compare the relative effectiveness of the two study therapies:

- (1) Count of relapses.
- (2) Time to first relapse.
- (3) Time to first confirmed disability worsening event. Worsening was defined as an increase of ≥ 1.5 EDSS steps if baseline EDSS was 0, or 1.0 if baseline EDSS was 1.0–5.5, or 0.5 steps if baseline EDSS was > 5.5, and sustained at all consecutive visits over ≥ 6 months (confirmation cannot be preceded by a relapse within 30 days).
- (4) Time to first confirmed disability improvement event. An improvement was defined as a decrease of 1.5 if baseline EDSS was 1.5, or 1.0 if baseline EDSS was 2.0–6.0, or 0.5 if baseline EDSS was > 6, sustained at all consecutive visits over ≥ 6 months.

The end of analyzed study or period (count of relapses) depended on the definition of right-censoring (see below).

### Assignment procedure: propensity score matching and weighting

In the present work, baseline was defined as the date of the start of the index therapy. To emulate the random assignment of treatments at baseline, PS [13, 27] was defined as the probability of being treated with natalizumab, conditional on the following baseline characteristics (based on expert opinion and prior analyses): sex, age, MS duration (from first MS symptoms to baseline), EDSS score, number of previous treatments, and, evaluated in the past 12 months: number of relapses, and the nature of clinical activity recorded (disability worsening only, relapses only, both or no clinical activity). Country was added as random effect. We estimated both the average treatment effect for the treated (ATT) which is the average treatment effect among those patients who were exposed to natalizumab, and the average treatment effect for the entire eligible population (ATE) [28]. One-to-one, greedy, nearest neighbor, random matching on PS was used, allowing for approximating ATT only [29]. Matching caliper values of 0.1 (used in the original studies), 0.2 (as recommended by literature [30]) and 0.02 standard deviations of the PS (to prioritize close matching) were used. Two weighting procedures were explored. First, using Inverse Probability of Treatment Weighting (IPTW), the weights for a treated patient and for a control are defined as ${w}_{i }=\frac{1}{{p}_{i}}$ and ${w}_{i}=\frac{1}{1-{p}_{i}}$, respectively, where ${p}_{i}$ is the PS for a patient $i$. In order to reduce issue due to extreme weights, the weights were stabilized by multiplication by the marginal probability of receiving the treatment actually received [31], referred to as sIPTW. Second, using odds [32], the weight for a treated patient is 1 and the weight for control is defined${w}_{i}=\frac{{p}_{i}}{1-{p}_{i}}$. Weighting with IPTW allows estimation of ATE while weighting by the odds allows estimation of ATT.

### Causal contrast of interest

Intention-to-treat analysis retained all matched or weighted patients in the group as initial treatment allocation regardless of their following exposure, until either the last data entry or the study outcome. Per-protocol analysis retained all matched or weighted patients until the date of treatment discontinuation (or the date of last data entry if it occurs earlier). Pairwise-censoring was used as a technique of censoring after matching. In each pair, study follow-up of both patients was censored when the follow-up of one of the two patients was censored. This approach prevented imbalance due to differential duration of follow-up in the matched groups.

### Sensitivity analysis without the positivity assumption

The primary analysis ensured that the positivity assumption was fulfilled by only including patients who commenced natalizumab or fingolimod after the more recent of the two therapies became available on 1^st^ January 2011. In a sensitivity analysis, all patients who commenced a study therapy were included, irrespective of the commencement date. Therefore, patients that were considered as ineligible in the primary analysis were included in this sensitivity analysis. Before 2011, MS patients had no chance to receive fingolimod, and could only started natalizumab; that is why the positivity assumption was violated.

### Statistical analysis

Characteristics of the patients included in the analyses as well as those excluded by the matching procedure were described – overall and by treatment groups, before and after PS matching/weighting. Standardized mean differences (SMD) or Mahalanobis distances were computed, with 10% considered to be an acceptable difference [33]. Incidence of relapses was evaluated using a negative binomial model, with an offset term for follow-up durations. The cumulative hazards of first relapse, first EDSS improvement and first EDSS worsening were studied using Cox proportional hazards models with robust estimation of variance [34]. The models were either weighted by sIPTW or odds, or matched on PS. A cluster term (generalized estimating equations with negative binomial distribution) or a frailty term (Cox models) for pair identifier was used. As the probability of disability worsening and improvement events is associated with the frequency of EDSS scores [35], models with time to disability outcomes were adjusted for annualized visit density. All analyses were conducted for both the intention-to-treat and the per-protocol causal contrasts. Analyses using matching were completed with and without pairwise-censoring. Table 1 gives an overview of all the analytical approaches considered in the present work. The analyses were performed using R-software (R 3.4.0).

**Table 1:** Overview of the analytical approaches used in the present work according to the outcomes

Outcome	PS method	Model
**Counts of relapses**^**a**^	Weighting^b^	Weighted negative binomial model of disease outcomes by treatment
	Matching^c^	Generalized estimating equations with negative binomial distribution and cluster for treatment status
**Time to first relapse**^**a**^	Weighting^b^	Weighted Cox model of disease outcomes by treatment
	Matching^c^	Frailty Cox model of disease outcomes by treatment
**Time to first EDSS worsening**^**a**^	Weighting^b^	Weighted Cox model of disease outcomes by treatment adjusted for visit density
	Matching^c^	Frailty Cox model of disease outcomes by treatment adjusted for visit density
**Time to first EDSS improvement**^**a**^	Weighting^b^	Weighted Cox model of disease outcomes by treatment adjusted for visit density
	Matching^c^	Frailty Cox model of disease outcomes by treatment adjusted for visit density

^a^Analyses were conducted in intention-to-treat, on treatment and pairwise-censoring (matching only) frameworks

^b^Two type of weights were considered (inverse probability weighting and weighting by the odds)

^c^Three values of calipers were considered (0.02, 0.1, 0.2)

## Results

### Patients’ characteristics

Overall, 5,148 patients were included in this study [10]; 1,989 (39%) were treated with natalizumab and 3,159 (61%) with fingolimod. Patient’s characteristics are described in Table 2 (overall median age at baseline: 37.7 years; median MS duration at baseline: 6.9 years). Most of the patients had a clinically active disease and 70% had a baseline EDSS score equal or greater than 2. Table 3 presents the median durations of follow-up (overall: 3.1 years (interquartile range (IQR): 2.0–4.5)). The median durations of natalizumab and fingolimod treatments were 2.00 (1.3–3.1) and 2.2 (1.2–3.6) years, respectively.

**Table 2:** Baseline characteristics of the overall study population, as well as the subgroups of patients unmatched and matched within different calipers

	**Overall**	**Matching with caliper = 0.1**		**Matching with caliper = 0.2**		**Matching with caliper = 0.02**	
	**ALL**	**Matched**	**Excluded**	**Matched**	**Excluded**	**Matched**	**Excluded**
	***N* = 5148**	***N* = 3258**	***N* = 1890**	***N* = 3278**	***N* = 1870**	***N* = 3232**	***N* = 1916**
**Sex**^**a**^							
Female	3698 (72%)	2342 (72%)	1356 (72%)	2352 (72%)	1346 (72%)	2332 (72%)	1366 (71%)
Male	1450 (28%)	916 (28%)	534 (28%)	926 (28%)	524 (28%)	900 (28%)	550 (29%)
**Age at treatment start**^**b**^	37.7 (30.1–44.7)	37.3 (30.1–44.3)	38.5 (31.8–45.6)	37.2 (30.1–44.4)	38.7 (31.8–45.4)	37.4 (30.2–44.5)	38.3 (31.5–45.1)
**MS duration at treatment start**^**b**^	6.9 (3.1–12.5)	6.4 (2.7–2.0)	7.9 (4.0–13.4)	6.4 (2.6–2.0)	7.9 (4.0–13.4)	6.3 (2.6–12.0)	8.1 (4.0–13.3)
**EDSS at treatment start**^**a**^							
Less than 2	1556 (30%)	782 (24%)	774 (41%)	810 (25%)	746 (40%)	789 (24%)	767 (40%)
Between 2 and 3.5	2384 (46%)	1609 (49%)	775 (41%)	1593 (49%)	791 (42%)	1588 (49%)	796 (42%)
4 or more	1208 (23%)	867 (27%)	341 (18%)	875 (27%)	33 (18%)	855 (26%)	353 (18%)
**Number of relapses in the previous 12 months**^**a**^							
0	1857 (36%)	1063 (33%)	794 (42%)	1085 (33%)	772 (41%)	1059 (33%)	798 (42%)
1	2021 (39%)	1290 (40%)	731 (39%)	1268 (39%)	753 (40%)	1276 (39%)	745 (39%)
2	975 (19%)	690 (21%)	285 (15%)	707 (22%)	268 (14%)	696 (22%)	279 (15%)
3 or more	295 (6%)	215 (7%)	80 (4%)	218 (7%)	77 (4%)	107 (7%)	94 (5%)
**Number of previous MS treatments**^**a**^							
0	836 (16%)	582 (18%)	254 (13%)	580 (18%)	256 (14%)	584 (18%)	252 (13%)
1	2559 (50%)	1594 (49%)	965 (51%)	1597 (49%)	962 (51%)	1558 (48%)	1001 (52%)
2	1187 (23%)	738 (23%)	449 (24%)	744 (23%)	443 (24%)	748 (23%)	439 (23%)
3 or more	566 (11%)	344 (11%)	222 (12%)	357 (11%)	209 (11%)	342 (11%)	224 (12%)
**MS activity in the previous 12 months**^**a**^							
None	1438 (28%)	776 (24%)	662 (35%)	782 (24%)	656 (35%)	764 (24%)	674 (35%)
Worsening	419 (8%)	287 (9%)	132 (7%)	303 (9%)	116 (6%)	295 (9%)	124 (6%)
Relapse	2159 (42%)	1395 (43%)	764 (40%)	1398 (43%)	761 (41%)	1397 (43%)	762 (40%)
Relapse and worsening	1132 (22%)	800 (25%)	332 (18%)	795 (24%)	337 (18%)	776 (24%)	356(19%)
**Data source**^**a**^							
MS Base	3293 (64%)	1874 (58%)	1419 (75%)	1882 (57%)	1411(75%)	1852 (57%)	1441 (75%)
DMSR	1444 (28%)	1167 (36%)	277 (15%)	1179 (36%)	265 (14%)	1153 (36%)	291 (15%)
OFSEP	411 (8%)	217 (7%)	194 (10%)	217 (7%)	194 (10%)	227 (7%)	184 (10%)

^a^N (%)

^b^Median (Quartiles)

**Table 3:** Follow-up duration according to the outcomes of interest (in years)

Outcome	Intention-to-treat analysis	Per-protocol analysis
Counts of relapses^a^	3.17 (2.01–4.59)	2.09 (1.24–3.41)
Time to first relapse^b^	3.11 [3.05; 3.18]	2.27 [2.21; 2.31]
Time to first EDSS worsening^b^	3.16 [3.10; 3.23]	2.11 [2.07; 2.16]
Time to first EDSS improvement^b^	3.20 [3,13; 3.27]	2.08 [2.02; 2.12]

^a^ Median (Quartiles) length of follow-up

^b^ Median [95% confidence interval] survival time of the reverse Kaplan–Meier, taking into account the length and the completeness of follow-up

### Patients’ characteristics after propensity score balancing procedures (matching and weighting)

**Fig. 1:**
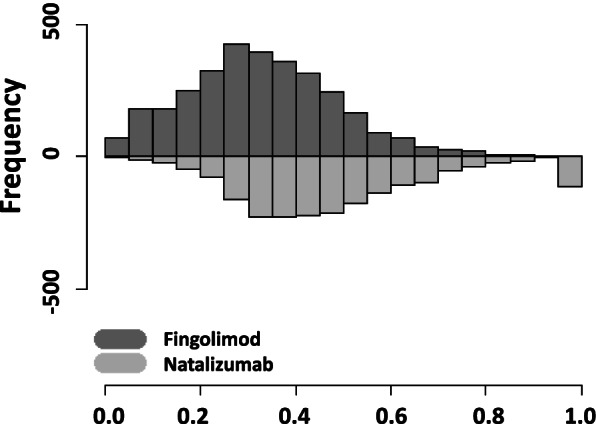
Distribution of propensity scores by treatment group (probability of being treated with natalizumab)

The distributions of PS showed a good overlap between the treatment groups, except in the tails (Fig. 1). The use of three caliper values for PS-matching led to three similar matched datasets (Table 2). The characteristics of the matched groups were comparable to the characteristics of the overall sample. The excluded patients tended to experience less disease activity. Table 4 presents patients’ characteristics by treatment group. Overall, 35% of patients treated with fingolimod had an EDSS score < 2 at treatment start while it was 22% in the group treated with natalizumab. The matching procedure improved the balance between the compared groups, except for the data source and the number of previous MS treatments.

**Table 4:** Characteristics at baseline according to treatment group in the overall population and when three matching calipers were used

	**Overall**			**Matching (caliper = 0.1)**			**Matching (caliper = 0.2)**			**Matching (caliper = 0.02)**		
	*N* = 5148			*N* = 3258			*N* = 3278			*N* = 3232		
	**natalizumab**	**fingolimod**	**SMD**^**c**^	**natalizumab**	**fingolimod**	**SMD**^**c**^	**natalizumab**	**fingolimod**	**SMD**^**c**^	**natalizumab**	**fingolimod**	**SMD**^**c**^
	*N* = 1989	*N* = 3159		*N* = 1629	*N* = 1629		*N* = 1639	*N* = 1639		*N* = 1616	*N* = 1616	
**Sex**^**a**^			4%			1%			2%			0.8%
Female	1451 (73%)	2247 (71%)		1175 (72%)	1167 (72%)		1183 (72%)	1169 (71%)		1169 (72%)	1163 (28%)	
Male	538 (27%)	912 (29%)		454 (28%)	462 (28%)		456 (28%)	470 (29%)		447 (28%)	453 (28%)	
**Age at treatment start**^**b**^ **MS duration at treatment**^**b**^	36.6 (29.3; 43.9) 6.3 (2.4; 11.8)	38.5 (31.6; 45.4) 7.4 (3.6; 13.0)	13% 13%	37.2 (29.8–44.4) 6.3 (2.3–12.2)	37.4 (30.3–44.2) 6.5 (2.9–11.8)	0.5% 2%	37.2 (29.7–44.4) 6.2 (2.3–12.2)	37.4 (30.3–44.3) 6.6 (2.9–11.9)	1% 2%	37.3 (29.7–44.4) 6.3 (2.3–12.2)	37.6 (30.5–44.5) 6.4 (2.8–11.9)	3% 2%
**EDSS at treatment start**^**a**^			32%			5%			9%			7%
2 or less	434 (22%)	1122 (35%)		374 (23%)	408 (25%)		377 (23%)	433 (26%)		372 (23%)	417(26%)	
Between 2 and 4	981 (49%)	1403 (44%)		822 (50%)	787 (48%)		826 (50%)	767 (47%)		813 (50%)	775 (48%)	
4 or more	574 (29%)	634 (20%)		433 (27%)	434 (27%)		436 (25%)	439 (27%)		431 (27%)	424 (26%)	
**Number of relapses in the previous 12 months**^**a**^			37%			8%			6%			6%
0	570 (29%)	1287 (41%)		543 (33%)	520 (32%)		545 (33%)	540 (33%)		541 (33%)	518 (32%)	
1	752 (38%)	1269 (40%)		620 (38%)	670 (41%)		623 (38%)	645 (39%)		618 (38%)	658 (41%)	
2	484 (24%)	491 (15%)		346 (21%)	344 (21%)		351 (22%)	356 (22%)		350 (22%)	346 (21%)	
3 or more	183 (9%)	112 (3%)		120 (7%)	95 (6%)		120 (7%)	98 (6%)		107 (7%)	94 (6%)	
**Number of previous MS treatments**^**a**^			17%			17%			17%			16%
0	401 (20%)	435 (14%)		334 (21%)	248 (15%)		337 (21%)	243 (15%)		334 (21%)	250 (15%)	
1	924 (46%)	1635 (52%)		742 (46%)	852 (52%)		746 (46%)	851 (52%)		732 (4%)	826 (51%)	
2	457 (23%)	730 (23%)		367 (23%)	371 (23%)		370 (23%)	374 (23%)		365 (23%)	383 (24%)	
3 or more	207 (10%)	359 (11%)		186 (11%)	158 (10%)		186 (11%)	171 (10%)		185 (11%)	157 (10%)	
**MS activity in the previous 12 months**^**a**^			29%			4%			2%			3%
None	410 (21%)	1028 (32%)		393 (24%)	383 (24%)		395 (24%)	387 (24%)		390 (24%)	374 (23%)	
Worsening	160 (8%)	259 (8%)		150 (9%)	137 (8%)		150 (9%)	153 (9%)		151(9%)	144 (9%)	
Relapse	886 (44%)	1273 (40%)		686 (42%)	709 (44%)		694 (42%)	704 (43%)		690 (43%)	707 (44%)	
Relapse and worsening	533 (27%)	599 (19%)		400 (25%)	400 (25%)		400 (24%)	395 (24%)		385 (24%)	391 (24%)	
**Data source**^**a**^			28%			18%			16%			12%
MS Base	1141 (57%)	2152 (68%)		949 (58%)	925 (57%)		957 (58%)	925 (56%)		935 (58%)	917 (57%)	
DMSR	607 (31%)	837 (26%)		607 (37%)	560 (34%)		607 (37%)	572 (35%)		593 (37%)	560 (35%)	
OFSEP	241 (12%)	170 (5%)		73 (4%)	144 (9%)		75 (5%)	142 (9%)		88 (5%)	139 (9%)	

^a^N (%)

^b^Median (Quartiles)

^c^*SMD* standardized mean differences or Mahalanobis distances between Natalizumab treated patients and Fingolimod treated patients

Table 5 presents patients’ characteristics by treatment group after weighting on sIPTW or odds. The treatment groups were well balanced, with SMD or Mahalanobis distances around 10% for all patient characteristics, except for the number of previous MS treatments, as natalizumab tended to be prescribed as first treatment more frequently than fingolimod. Exposure following the study therapy is shown in Table S1.

**Table 5:** Characteristics at baseline by treatment group in the overall study sample, and cohorts weighted on sIPTW and odds

	**Unweighted**			**Weighting using sIPTW**			**Weighting using the odds**		
	*N* = 5148								
	**natalizumab**	**fingolimod**	**SMD**^**c**^	**natalizumab**	**fingolimod**	**SMD**^**c**^	**natalizumab**	**fingolimod**	**SMD**^**c**^
	*N* = 1989	*N* = 3159							
**Sex**^**a**^			4%			1%			1%
Female	1451 (73%)	2247 (71%)		71%	72%		73%	73%	
Male	538 (27%)	912 (29%)		29%	28%		27%	27%	
**Age at treatment start**^**b**^	36.6 (29.3; 43.9)	38.5 (31.6; 45.4)	13%	37.6 (30.4–45.2)	37.9 (30.7–44.8)	2%	36.6 (29.3–43.9)	36.8 (29.8–43.9)	1%
**MS duration at treatment**^**b**^	6.3 (2.4; 11.8)	7.4 (3.6; 13.0)	13%	6.8 (2.7–12.9)	7.0 (3.2–12.4)	2%	6.2 (2.4–11.8)	6.2 (2.7–11.6)	1%
**EDSS at treatment start**^**a**^			32%			12%			7%
2 or less	434 (22%)	1122 (35%)		26%	31%		22%	23%	
Between 2 and 4	981 (49%)	1403 (44%)		50%	45%		49%	46%	
4 or more	574 (29%)	634 (20%)		24%	24%		29%	31%	
**Number of relapses in the previous 12 months**^**a**^			37%			4%			3%
0	570 (29%)	1287 (41%)		35%	36%		29%	29%	
1	752 (38%)	1269 (40%)		39%	39%		38%	37%	
2	484 (24%)	491 (15%)		20%	18%		24%	23%	
3 or more	183 (9%)	112 (3%)		6%	6%		9%	10%	
**Number of previous MS treatments**^**a**^			17%			15%			14%
0	401 (20%)	435 (14%)		19%	14%		20%	15%	
1	924 (46%)	1635 (52%)		46%	52%		46%	52%	
2	457 (23%)	730 (23%)		23%	23%		23%	23%	
3 or more	207 (10%)	359 (11%)		12%	11%		10%	10%	
**MS activity in the previous 12 months**^**a**^			29%			3%			3%
None	410 (21%)	1028 (32%)		26%	28%		21%	20%	
Worsening	160 (8%)	259 (8%)		8%	8%		8%	9%	
Relapse	886 (44%)	1273 (40%)		42%	42%		44%	43%	
Relapse and worsening	533 (27%)	599 (19%)		23%	22%		27%	27%	
**Data source**^**a**^			28%			1%			8%
MS Base	1141 (57%)	2152 (68%)		30%	29%		57%	53%	
DMSR	607 (30%)	837 (26%)		62%	62%		30%	34%	
OFSEP	241 (12%)	170 (5%)		8%	8%		12%	13%	

^a^N (%)

^b^Median (Quartiles)

^c^*SMD* standardized mean differences and Mahalanobis distance between natalizumab treated patients and fingolimod treated patients

### Comparison of effectiveness between natalizumab and fingolimod

**Fig. 2:**
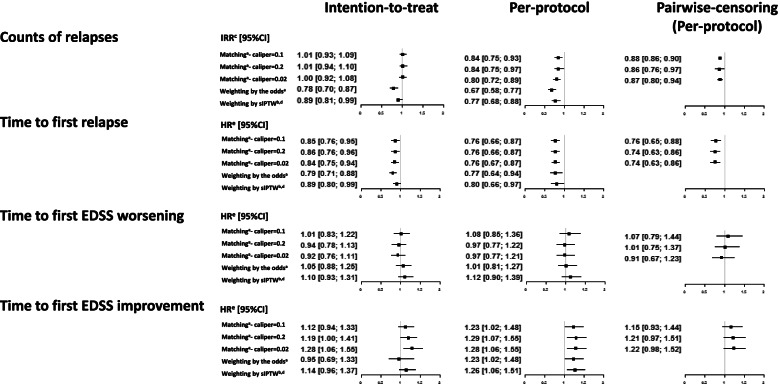
Estimated treatment effects for the 4 outcomes, 3 matching and 2 weighting strategies and 2 causal effects, with and without pairwise censoring in matched cohorts

Figure 2 summarises the results of all comparative analyses. While the estimated 95% confidence intervals of the estimated differences between natalizumab and fingolimod largely overlapped in all analyses, some variation in point estimates was observed.

With a few exceptions, the results of the analyses with matching and weighting led to the same conclusions, i.e., superiority of natalizumab (for relapse outcomes and EDSS improvement) or no evidence of difference (for EDSS worsening). Inconsistencies were observed mainly in the intention-to-treat frameworks, for relapse counts and first EDSS improvement. Weighting by the odds (ATT) tended to provide lower point estimates and similar margins of error of the relative effect compared to weighting by sIPTW (ATE). The value of the matching caliper did not influence the magnitude of the estimated differences.

Most of the variability in the estimates was linked to the causal contrast. The intention-to-treat paradigm led to less stable results, especially for the count of relapses and first EDSS improvement. For all outcomes except time to first EDSS worsening, the intention-to-treat analyses underestimated the differences between the therapies in comparison to per-protocol analyses with or without pairwise-censoring. Per-protocol analyses and pairwise-censored analyses returned similar point estimates, even though the margin of error varied. In the pairwise-censored analyses, confidence intervals were relatively smaller for relapse counts but larger for the disability outcomes compared to the per-protocol analysis.

### Sensitivity analysis: positivity assumption

To test the effect of violation of the positivity assumption, 7,118 patients were included irrespectively of the date of their treatment start, of whom 3,726 were treated with natalizumab. The other baseline characteristics were similar to those of the main cohort (Table S3). The PS distribution was left-skewed in patients who commenced natalizumab before fingolimod became available (Figure S1). Using weighting, the comparison of the treatment effects on relapses was similar to the main analysis (Table 6). However, the point estimates for the difference in the treatment effects on EDSS worsening were substantially lower than in the primary analysis, although confidence intervals overlapped. When matching was used, the estimates for EDSS outcomes were less influenced by the violation of the positivity assumption. Nevertheless, the estimates of the differences between treatment effects on relapses were substantially inflated when the assumption was violated, especially for the intention-to-treat causal effect.

**Table 6:** Comparison of treatment effect on relapses and disability violating the positivity assumption

		**Intention to treat**	**Per-protocol**
**Counts of relapses**	**IRR**^**c**^** [95%CI]**		
* ATT*^*a*^	**Matching- caliper = 0.1**	1.49 [1.36; 1.65]	0.95 [0.86; 1.04]
* ATE*^*b*^	**Weighting by sIPTW**^**d**^	0.92 [0.85; 0.99]	0.78 [0.70; 0.86]
**Time to first relapse**	**HR**^**e**^** [95%CI]**		
* ATT*^*a*^	**Matching- caliper = 0.1**	0.93 [0.79, 1.09]	0.82 [0.72, 0.92]
* ATE*^*b*^	**Weighting by sIPTW**^**d**^	0.91 [0.83; 1.00]	0.92 [0.79; 1.08]
**Time first EDSS worsening**	**HR**^**e**^** [95%CI]**		
* ATT*^*a*^	**Matching- caliper = 0.1**	0.92 [0.78, 1.08]	0.93 [0.75, 1.14]
* ATE*^*b*^	**Weighting by sIPTW**^**d**^	0.88 [0.65; 1.20]	1.02 [0.77; 1.36]
**Time to first EDSS improvement**	**HR**^**e**^** [95%CI]**		
* ATT*^*a*^	**Matching- caliper = 0.1**	1.07 [0.91, 1.26]	1.23 [1.03, 1.47]
* ATE*^*b*^	**Weighting by sIPTW**^**d**^	0.89 [0.66; 1.19]	1.01 [0.76; 1.35]

^a^Average treatment effect for treated

^b^Average treatment effect for the entire population

^c^Incidence Rate Ratio

^d^Stabilized inverse probability of treatment weighting

^e^Hazard ratio

## Discussion

In this empirical study conducted on a complex chronic neurological condition, with long-term follow-up data, several non-linear outcomes and well powered dataset, most of the methodological choices (PS matching/weighting, caliper values, weighting on IPTW vs. odds, and pairwise censoring) resulted in consistent overall conclusions, in accordance with two of the three original studies [5, 6], the pooled analysis [11] and a recent French head-to-head prospective study [36]. In a longitudinal observational study conducted over the long-term in the presence of frequent changes of therapy, an intention-to-treat causal contrast tends to be associated with more variability in the observed effects than a per-protocol contrast. Importantly, violation of the positivity assumption demonstrated the most pronounced negative effect on the consistency of reported results.

### Propensity score to control indication bias

Among the four methods using PS, matching and weighting have shown a superior performance to adjustment and stratification in achieving balance on baseline characteristics [37], reduction of bias and estimation of variance [38–40]. Therefore, we restricted our present work to PS matching and weighting. The results of the weighting and matching procedures were consistent, confirming that both methods performed well in sufficiently powered data sets and correctly specified models. The width of the matching caliper did not have much influence on the consistency of the results, confirming that 0.2 is a sufficiently conservative caliper, as previously reported [30]. The only detectable systematic variability was noted for the type of estimated effect, with the magnitude of the ATE effect trending towards higher values for relapse incidence and time to first relapse.

The matched study sample corresponds to an overlap between the fingolimod- and the natalizumab-treated target populations, with inclusion of comparable cases and exclusion of cases outside the common distribution of the PS (ATT effect of interest). Such reductions in sample size may lead one to study a very specific sub-population and, so, impact the precision and the generalizability of the results [41]. An IPTW-weighted sample is closer to the entire study population, especially where ATE is the effect of interest. It is therefore not surprising, given that the use of natalizumab and fingolimod in MS differs in clinical settings, that we have observed differences in the point estimates obtained with the matched and weighted analyses. Weighting could potentially be subject to influential cases with extreme weights, which are excluded from matching, as they fall outside of the central portion of the PS distribution [42]. In this work, we used stabilized weights to mitigate the risk of influential cases, as an alternative to weight trimming or truncation [33].

### Management of censoring

In the present study, most irregularities were related to the intention-to-treat causal contrast, which resulted in less stable and often deflated estimates than the per-protocol analysis. These fluctuations were more pronounced for the outcomes defined as counts of events and time to medium-term events (first disability worsening or improvement) than for time to short-term events (first relapse). The intention-to-treat evaluates the association with the outcome, irrespective of treatment status over-time, and addresses the question of the effect of treatment decision, irrespective of further persistence on the assigned therapy. Therefore, such an approach leads to conservative estimates, which explains the observed overall deflation of effect sizes in comparison to the per-protocol approach and the minimum impact on short-term outcomes.

On the other hand, patients and neurologists may be more interested in a per-protocol effect, which estimates the effect of an intervention while being adhered to. However, a per-protocol treatment effect can be inflated by attrition bias and informed censoring, especially when one of the compared interventions is a-priori perceived as being more effective [43]. This would lead to the selection of “treatment responders”, because patients who respond well to treatment are more likely to remain treated than non-responders [44]. In addition, the per-protocol requirement of adherence to treatment may introduce additional selection bias, which may limit generalizability of conclusions [45], whereas the intention-to-treat approach preserves the balance established at baseline. A pairwise-censoring procedure can be combined with either causal contrast. Its purpose is to sustain the balance between the matched cohorts even when censoring / treatment cessation is systematically different between the compared groups. This sustained balance is achieved at the expense of loss of part of study follow-up due to right-censoring of the paired cases. However, in the present empirical analysis, per-protocol and pairwise-censored analyses led to similar conclusions and point estimates. The observed increase in the margin of error in pairwise-censored analysis suggests some loss of power. Marginal structural models with IPTWs accounting for the probability of censoring may provide a more efficient solution, as they do not lead to loss of follow-up information [46–48].

### Positivity assumption

The positivity assumption can be objectively assessed in several steps. First, the definition of study timeline and area should be such as both treatments are available to all included patients. Second, the common support of PS distribution in the two groups needs to be established [31]. In our main analysis, these two steps confirmed that the positivity assumption was met. To examine the importance of the positivity assumption, in a different analysis, we allowed inclusion of patients before one of the studied therapies (fingolimod) became available. This included more natalizumab-treated patients from a time period when the probability of exposure to fingolimod was zero. The results of this analysis showed the most pronounced variability and the largest deviation from the primary analysis. Therefore, in a sufficiently powered longitudinal dataset, non-zero probability of exposure to both compared therapies at all baseline time-points is the most important aspect of methodological considerations explored in this study.

### Limitations

Through consistency and exchangeability assumptions, it is assumed that there were no unmeasured confounders. Nevertheless, our study was limited by incomplete MRI data, while MRI activity is a known prognostic factor in MS [49]. Reassuringly, two of our three previous studies that accounted for MRI at treatment start showed results consistent with our primary analysis [5, 6].

In addition, heterogeneity of data in multisite registries (with potential differences in therapeutic practices, health care systems and treatment access) may increase variance of the associations between treatments and outcomes [50]. On the other hand, heterogeneity that is representative of clinical use of the compared therapies extends generalizability of the results. We have mitigated the potential heterogeneity in the present dataset by including country as a random term in the PS modeling.

Finally, this study did not attempt to compare the efficiency and robustness of different analytical methods, as this can be done only with simulation studies. Instead, we have focused on the evaluation of practical methodological questions in the context of a specific clinical choice.

## Conclusion

This empirical study provides practical insights into the effects of several methodological choices on the estimates of the difference between two therapies in the context of a chronic neurological disease, in a sufficiently powered analysis and correctly specified models. Our results lead us to conclude that methodological considerations such as PS matching/weighting and their specifications, causal contrast and management of censoring have a negligible effect on the overall analyses, given that the model assumptions are met. The choice between ATT or ATE as the preferred approach should be driven by the clinical question of interest. In our clinical example, when both treatments can be prescribed to patients with relapsing–remitting MS following similar rules, there is no apparent reason to restrict the analysis to the natalizumab- or the fingolimod-treated patients, and ATE may be the preferred estimator of interest.

A recent review highlighted the good practice in the use and reporting of PS in MS [41]. While methodological choices in observational studies remain challenging, our present work illustrates the priorities for methodological aspects of PS-based analyses of comparative treatment effectiveness in large registries.

## Supplementary Information

**Additional file 1: Figure S1. **Propensity score distribution without the positivity assumption. **Table S1. **Treatment exposure after natalizumab or fingolimod start during the follow-up. **Table S2. **Baseline characteristics of the unmatched cohorts by treatment group. **Table S3. **Baseline characteristics of cohort violating the positivity assumption.  

## Data Availability

OFSEP: The individual data from the present study can be obtained upon request and after validation from the OFSEP scientific committee (see website: http://www.ofsep.org/en/dataaccess). MSBase: MSBase is a data processor, and warehouses data from individual principal investigators who agree to share their datasets on a project-by-project basis. Each principal investigator will need to be approached individually for permission to access the datasets. DMSR: Anonymized data will be shared on request from any qualified researcher under approval from the Danish Data Protection Agency.

## Abbreviations

ATT: Average Treatment effect for the Treated
ATE: Average Treatment effect for the Entire eligible population
EDMUS: European Database for Multiple Sclerosis
EDSS: Expanded Disability Status Scale
IPTW: Inverse Probability of Treatment Weighting
MS: Multiple Sclerosis
PS: Propensity Score
RRMS: Relapsing Remitting Multiple Sclerosis
sIPTW: Stabilized Inverse Probability of Treatment Weighting
SMD: Standardized Mean Differences
