# Supplementary material for: Impact of methodological choices in comparative effectiveness studies: application in natalizumab versus fingolimod comparison among patients with multiple sclerosis
Source: BMC Med Res Methodol. 2022 May 30;22:155. doi: 10.1186/s12874-022-01623-8 (PMC9150358; doi:10.1186/s12874-022-01623-8)
Supplement: Supplementary file 1 — Additional file 1: Figure S1. Propensity score distribution without the positivity assumption. Table S1. Treatment exposure after natalizumab or fingolimod start during the follow-up. Table S2. Baseline characteristics of the unmatched cohorts by treatment group. Table S3. Baseline characteristics of cohort violating the positivity assumption. [file 12874_2022_1623_MOESM1_ESM.docx]

**Supplementary materials**

**Table S1: Treatment exposure after natalizumab or fingolimod start during the follow-up.**

| **Fingolimod-treated patients**  **(N= 3159)** | |  | **Natalizumab-treated patients**  **(N= 1989)** | |
| --- | --- | --- | --- | --- |
| **Sequence^a^** | **N (%)** |  | **Sequence^a^** | **N (%)** |
| Unknown due to ongoing fingolimod or loss of follow-up | 2577 (81%) |  | Unknown due to ongoing natalizumab or loss of follow-up | 1311 (66%) |
| Natalizumab | 222 (7%) |  | Fingolimod | 393 (20%) |
| Fingolimod | 93 (3%) |  | Natalizumab | 101 (5%) |
| Dimethyl Fumarate | 42 (1%) |  | Dimethyl Fumarate | 23 (1%) |
| Teriflunomide | 29 (0.9%) |  | Glatiramer acetate | 14 (0.7%) |
| Interferon | 15(0.5%) |  | Fingolimod, Natalizumab | 12 (0.6%) |
| Glatiramer acetate | 15 (0.5%) |  | Rituximab | 11 (0.5%) |

^a^sequence observed for 0.5% or more patients

|  | **Overall** | | **Unmatched – caliper=0.1**  N=1890 | | **Unmatched – caliper=0.2**  N=1870 | | **Unmatched – caliper=0.02**  N=1916 | | |
| --- | --- | --- | --- | --- | --- | --- | --- | --- | --- |
|  | N=5148 | |  |  |  |  |  |  |  |
|  | **natalizumab** | **fingolimod** | **natalizumab** | **fingolimod** | **natalizumab** | **fingolimod** | **natalizumab** | **fingolimod** |  |
|  | N=1989 | N=3159 | N=360 | N=1530 | N=350 | N=1520 | N=373 | N=1543 |  |
| **Sex^a^** |  |  |  |  |  |  |  |  |  |
| Female | 1451 (73%) | 2247 (71%) | 276 (77%) | 1356 (71%) | 268 (77%) | 1078 (71%) | 282 (76%) | 1084 (70%) |  |
| Male | 538 (27%) | 912 (29%) | 84 (23%) | 534 (29%) | 82 (23%) | 442 (29%) | 91 (24%) | 459 (30%) |  |
| **Age at treatment start^b^** | 36.6 (29.3-43.9) | 38.5 (31.6-45.4) | 37.2 (29.8- 44.4) | 37.4(30.3-44.2) | 37.2 (29.7-44.4) | 37.3 (30.3-44.3) | 37.3 (29.8-44.4) | 37.6 (30.5-44.5) |  |
| **MS duration at treatment^b^** | 6.3 (2.4-11.8) | 7.4 (3.6-13.0) | 6.3 (2.3-12.2) | 6.5(2.9-11.8) | 6.2 (2.3-12.2) | 6.6 (2.9-11.9 | 6.3 (2.33-12.2) | 6.4 (3.8-11.9) |  |
| **EDSS at treatment start^a^** |  |  |  |  |  |  |  |  |  |
| 2 or less | 434 (22%) | 1122 (35%) | 60 (17%) | 714 (47%) | 57 (16%) | 689 (45%) | 62 (17%) | 705 (46%) |  |
| Between 2 and 4 | 981 (49%) | 1403 (44%) | 159 (44%) | 616 (40%) | 155 (44%) | 636 (42%) | 168 (45%) | 628 (41%) |  |
| 4 or more | 574 (29%) | 634 (20%) | 141 (39%) | 200 (13%) | 138 (39%) | 195 (13%) | 143 (38%) | 210 (14%) |  |
| **Number of relapses in the previous 12 months^a^** |  |  |  |  |  |  |  |  |  |
| 0 | 570 (29%) | 1287 (41%) | 27 (8%) | 767 (50%) | 25 (7%) | 747 (49%) | 29 (8%) | 769 (50%) |  |
| 1 | 752 (38%) | 1269 (40%) | 132 (37%) | 599 (39%) | 129 (37%) | 624 (41%) | 134 (36%) | 611 (40%) |  |
| 2 | 484 (24%) | 491 (15%) | 138 (38%) | 147 (10%) | 133 (38%) | 135 (9%) | 134 (36%) | 145 (9%) |  |
| 3 or more | 183 (9%) | 112 (3%) | 63 (18%) | 17 (1%) | 63 (18%) | 14 (1%) | 76 (20%) | 18 (1%) |  |
| **Number of previous treatment^a^** |  |  |  |  |  |  |  |  |  |
| 0 | 401 (20%) | 435 (14%) | 67 (19%) | 187 (12%) | 64 (18%) | 192 (13%) | 67 (18%) | 185 (12%) |  |
| 1 | 924 (46%) | 1635 (52%) | 182 (51%) | 783 (51%) | 178 (51%) | 784 (52%) | 192 (51%) | 809 (52%) |  |
| 2 | 457 (23%) | 730 (23%) | 90 (25%) | 359 (23%) | 87 (25%) | 356 (23%) | 92 (25%) | 347 (22%) |  |
| 3 or more | 207 (10%) | 359 (11%) | 21 (6%) | 201 (13%) | 21 (6%) | 188 (12%) | 22 (6%) | 202 (13%) |  |
| **MS activity in the previous 12 months^a^** |  |  |  |  |  |  |  |  |  |
| None | 410 (21%) | 1028 (32%) | 17 (5%) | 645 (42%) | 15 (4%) | 641 (42%) | 20 (5%) | 654 (42%) |  |
| Worsening | 160 (8%) | 259 (8%) | 10 (3%) | 122 (8%) | 10 (3%) | 106 (7%) | 9 (2%) | 115 (7%) |  |
| Relapse | 886 (44%) | 1273 (40%) | 200 (56%) | 564 (37%) | 192 (55%) | 569 (37%) | 196 (53%) | 566 (37%) |  |
| Relapse and worsening | 533 (27%) | 599 (19%) | 133 (37%) | 199 (13%) | 133 (38%) | 204 (13%) | 148 (40%) | 208 (13%) |  |
| **Data sources^a^** |  |  |  |  |  |  |  |  |  |
| MS Base | 1141 (57%) | 2152 (68%) | 192 (53%) | 1227 (80%) | 184 (53%) | 1227 (81%) | 206 (55%) | 1235 (80%) |  |
| DMSR | 607 (31%) | 837 (26%) | 0 (0%) | 277 (18%) | 0 (0%) | 265 (17%) | 14 (4%) | 277 (18%) |  |
| OFSEP | 241 (12%) | 170 (5%) | 168 (47%) | 26 (2%) | 166 (47%) | 28 (2%) | 153 (41%) | 31 (2%) |  |
| ^a^N (%); ^b^Median (Quartiles); ^c^SMD: standardized mean differences between Natalizumab treated patients and Fingolimod treated patients | | | | | | | | |  |

**Table S2: Baseline characteristics of the unmatched cohorts by treatment group**

**Table S3: Baseline characteristics of cohort violating the positivity assumption**

|  | **Overall**  N= 7118 | | | | **Weighting using sIPTW** | | | **Matching – caliper=0.1** | | |
| --- | --- | --- | --- | --- | --- | --- | --- | --- | --- | --- |
|  |  |  |  |  |  | | |  | | |
|  | **All** | **NAT** | **FTY** | **SMD^c^** | **NAT** | **FTY** | **SMD^c^** | **NAT** | **FTY** | **SMD^c^** |
|  |  | N=3726 | N=3392 |  |  |  |  |  |  |  |
| **Sex^a^** |  |  |  |  |  |  |  |  |  |  |
| Female | 5170 (74%) | 2745 (74%) | 2425 (71%) | 5% | 72% | 73% | 2% | 72% | 72% | 1% |
| Male | 1948 (27%) | 981 (26%) | 967 (28%) |  | 28% | 27% |  | 28% | 28% |  |
| **Age at treatment start^b^** | 37.5 (30.5-44.5) | 36.7 (29.5-43.7) | 38.5 (31.6-45.5) | 13% | 37.5 (30.3-45.0) | 37.4 (30.2-44.5) | 3% | 36.9 (29.7-44.2) | 38.0 (30.9-44.7) | 6% |
| **MS duration at treatment^b^** | 7.3(3.4-12.7) | 7.0 (3.1-12.4) | 7.5 (3.7-13.1) | 7% | 7.3 (3.2-12.9) | 7.0 (3.2-12.5) | 3% | 6.6 (2.6-12.2) | 7.19 (3.3-12.6) | 7% |
| **EDSS at treatment start^a^** |  |  |  | 41% |  |  | 9% |  |  | 9% |
| 2 or less | 1905 (27%) | 718 (19%) | 1187 (35%) |  | 24% | 28% |  | 38% | 42% |  |
| Between 2 and 4 | 3196 (45%) | 1700 (46%) | 1496 (44%) |  | 47% | 43% |  | 32% | 31% |  |
| 4 or more | 2017 (28%) | 1308 (35%) | 709 (21%) |  | 29% | 29% |  | 31% | 27% |  |
| **Number of relapses in the previous 12 months^a^** |  |  |  | 56% |  |  | 3% |  |  | 4% |
| 0 | 2193 (31%) | 820 (22%) | 1373 (41%) |  | 31% | 32% |  | 30% | 31% |  |
| 1 | 2777 (39%) | 1407 (38%) | 1370 (40%) |  | 39% | 38% |  | 38% | 42% |  |
| 2 | 1519 (21%) | 990 (27%) | 529 (16%) |  | 21% | 21% |  | 22% | 21% |  |
| 3 or more | 629 (9%) | 509 (14%) | 120 (3%) |  | 9% | 9% |  | 9% | 5% |  |
| **Number of previous treatment^a^** |  |  |  | 10% |  |  | 6% |  |  | 14% |
| 0 | 1014 (14%) | 548 (15%) | 466 (14%) |  | 16% | 14% |  | 19% | 15% |  |
| 1 | 3380 (48%) | 1686 (45%) | 1694 (50%) |  | 46% | 48% |  | 45% | 49% |  |
| 2 | 1702 (24%) | 909 (24%) | 793 (23%) |  | 24% | 24% |  | 24% | 23% |  |
| 3 or more | 1022 (14%) | 583 (16%) | 439 (13%) |  | 15% | 14% |  | 12% | 12% |  |
| **MS activity in the previous 12 months^a^** |  |  |  | 43% |  |  | 3% |  |  | 4% |
| None | 1682 (24%) | 594 (16%) | 1088 (32%) |  | 23% | 24% |  | 22% | 23% |  |
| Worsening | 511 (7%) | 226 (6%) | 285 (8%) |  | 7% | 8% |  | 8% | 8% |  |
| Relapse | 3151 (44%) | 1779 (48%) | 1372 (40%) |  | 44% | 44% |  | 44% | 45% |  |
| Relapse and worsening | 1774 (25%) | 1127 (30%) | 647 (19%) |  | 25% | 24% |  | 25% | 23% |  |
| **Register^a^** |  |  |  |  |  |  | 1% |  |  | 34% |
| MS Base | 4286 (60%) | 1935 (52%) | 2351 (69%) | 70% | 59% | 59% |  | 69% | 64% |  |
| DMSR | 1466 (20%) | 617 (16%) | 849 (25%) |  | 22% | 22% |  | 30% | 28% |  |
| OFSEP | 1366 (19%) | 1174 (31%) | 192 (6%) |  | 19% | 19% |  | 1% | 9% |  |

^a^N (%); ^b^Median (Quartiles); ^c^SMD: standardized mean differences between Natalizumab treated patients and Fingolimod treated patients

**Figure S1: Propensity score distribution without the positivity assumption**

***
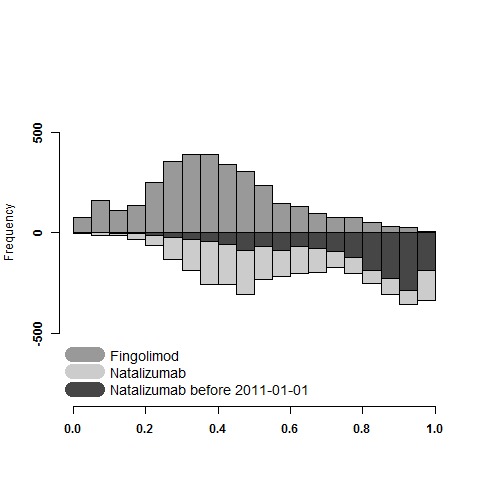
***
